# Supplementary material for: Matrix stiffness induces endothelial network senescence
Source: bioRxiv. 2025 Oct 6:2025.10.05.680536. Preprint. [Version 1] doi: 10.1101/2025.10.05.680536 (PMC12632338; doi:10.1101/2025.10.05.680536)
Supplement: Supplement 1 [file NIHPP2025.10.05.680536v1-supplement-1.pdf]

bioRxiv preprint doi: https://doi.org/10.1101/2025.10.05.680536; this version posted October 6, 2025. The copyright holder for this preprint (which was not certified by peer review) is the author/funder, who has granted bioRxiv a license to display the preprint in perpetuity. It is made available under aCC-BY 4.0 International license.

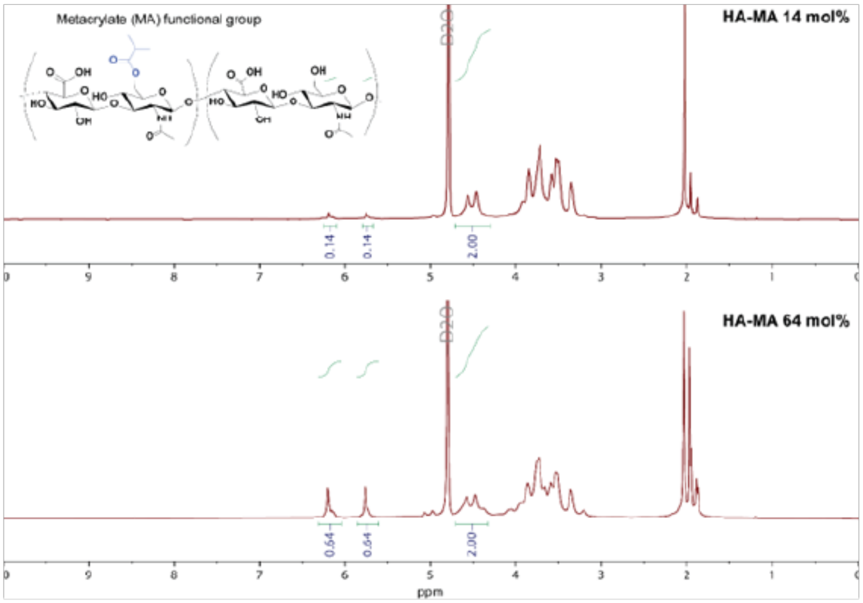

Figure S1: ^{1}H NMR spectra for HA-MA low (14 mol%, top) and high (64 mol%, bottom) in D_2O

bioRxiv preprint doi: https://doi.org/10.1101/2025.10.05.680536; this version posted October 6, 2025. The copyright holder for this preprint (which was not certified by peer review) is the author/funder, who has granted bioRxiv a license to display the preprint in perpetuity. It is made available under aCC-BY 4.0 International license.

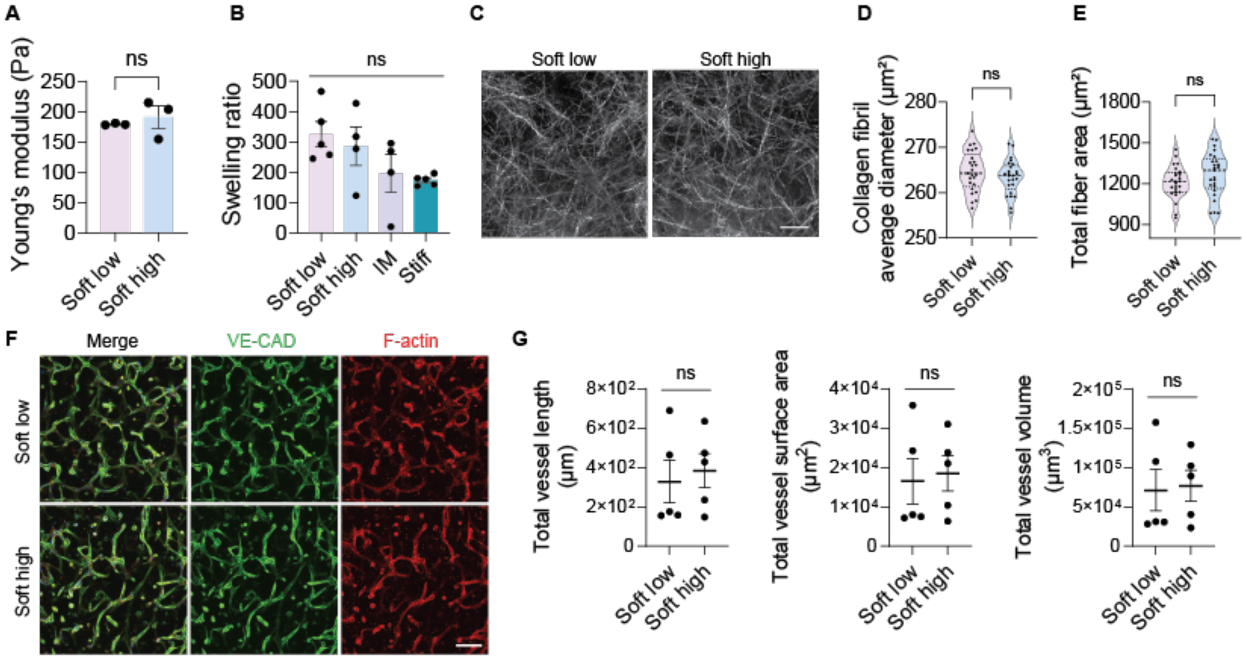

Figure S2: Hydrogel characterization of two Soft formulations prepared with varying methacrylate incorporation on HA. (A) Young's modulus measured by rheometry. (B) Equilibrium swelling ratio. (C) Representative confocal reflectance images showing comparable fibrillar microstructure (scale bar: 20 µm) as quantified in (D) by collagen fibril average diameter and in (E) by total fiber area. N=5 with 5 fields of images in each. (F) Representative maximum intensity projections of confocal z-stack showing microvascular networks in Soft low and Soft high hydrogels (Scale bar: 100 µm). (G) Quantification showing comparable microvascular network formation in terms of total vessel length, surface area, and volume.

	Collagen-MA Conc. (mg/mL)	HA-MA Conc. (mg/mL)	Degree of Methacrylate Substitution on HA (%)	Photoinitiator Exposure on Day 2	Light Exposure on Day 2	Young's Modulus (Pa)	Vascular Networks
**Soft low**	2.5	0.25	14	+	-	179.30 ± 0.92	+++
**Soft high**	2.5	0.25	64	+	-	191.17 ± 18.58	+++
**IM**	2.5	0.25	14	+	+	274.26 ± 5.72	++
**Stiff**	2.5	0.25	64	+	+	408.32 ± 11.02	+

Table S1. Summary of hydrogel formulations and secondary crosslinking parameters used to model dynamic matrix stiffening. Conditions vary by methacrylation level and light exposure to achieve a tunable range of stiffness, as reflected in Young's modulus measurements.

bioRxiv preprint doi: https://doi.org/10.1101/2025.10.05.680536; this version posted October 6, 2025. The copyright holder for this preprint (which was not certified by peer review) is the author/funder, who has granted bioRxiv a license to display the preprint in perpetuity. It is made available under aCC-BY 4.0 International license.

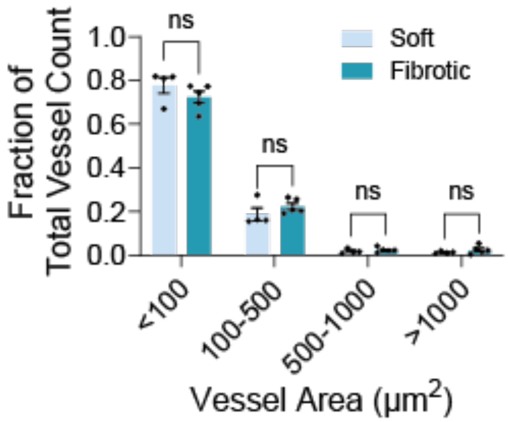

Fig S3. Quantification of endothelial vessel area distribution in soft vs. fibrotic breast capsule tissues. Bar graph showing the fraction of the total vessel count across four vessel area categories. Vessels were quantified from immunofluorescence image based on CD31 staining. No significant differences were observed between groups.

bioRxiv preprint doi: https://doi.org/10.1101/2025.10.05.680536; this version posted October 6, 2025. The copyright holder for this preprint (which was not certified by peer review) is the author/funder, who has granted bioRxiv a license to display the preprint in perpetuity. It is made available under aCC-BY 4.0 International license.

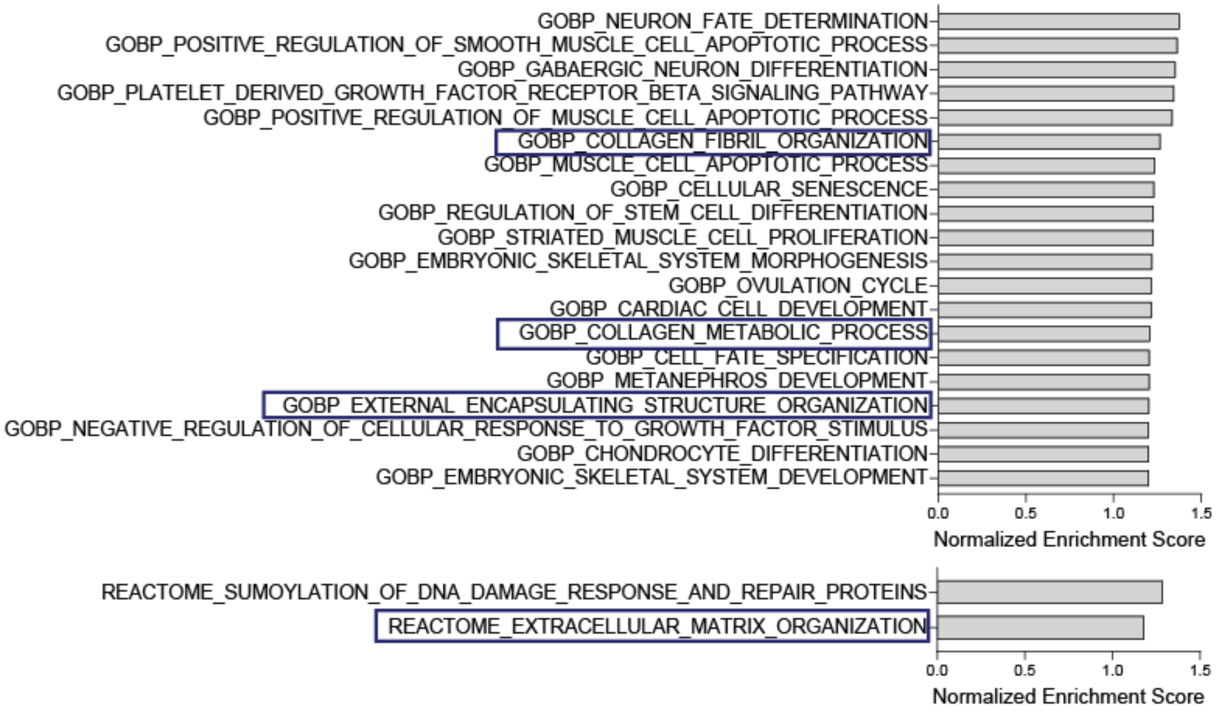

Fig S4. Gene Set Enrichment Analysis (GSEA) of p16$^+$ ECs showing enrichment of pathways (adjusted $p < 0.05$) related to collagen fibril organization, collagen metabolism, and extracellular organization.

bioRxiv preprint doi: https://doi.org/10.1101/2025.10.05.680536; this version posted October 6, 2025. The copyright holder for this preprint (which was not certified by peer review) is the author/funder, who has granted bioRxiv a license to display the preprint in perpetuity. It is made available under aCC-BY 4.0 International license.

Table S2. Forward and reverse primers used for qPCR analysis.

Gene	Forward Primer (5'-3")	Reverse Primer (5'-3")
GAPDH	ACAACTTTGGTATCGTGGAAGG	GCCATCACGCCACAGTTTC
CDKN1A	ACTCTCAGGGTCGAAAACGG	GATGTAGAGCGGGCCTTTGA
CDKN2A	ATGGAGCCTTCGGCTGACTGGC	CTGCCCATCATCATGACCTGGA
Notch1	CACTGTGGGCGGGTCC	GTTGTATTGGTTCGGCACCAT
JAG1	GACTCATCAGCCGTGTCTCA	TGGGGAACACTCACACTCAA

bioRxiv preprint doi: https://doi.org/10.1101/2025.10.05.680536; this version posted October 6, 2025. The copyright holder for this preprint (which was not certified by peer review) is the author/funder, who has granted bioRxiv a license to display the preprint in perpetuity. It is made available under aCC-BY 4.0 International license.

JAG2	GGTCGTACTTGCACTCACAATACC	GTAGCAAGGCAGAGGGTTGC
HEY1	GCTGGTACCCAGTGCTTTTGAG	TGCAGGATCTCGGCTTTTTCT
Dll4	GAAGTGGACTGTGGCCTGGACAAGT	TCGCTGATATCCGACACTCTGGCT
JUN	CCTTGAAAGCTCAGAACTCGGAG	TGCTGCGTTAGCATGAGTTGGC

bioRxiv preprint doi: https://doi.org/10.1101/2025.10.05.680536; this version posted October 6, 2025. The copyright holder for this preprint (which was not certified by peer review) is the author/funder, who has granted bioRxiv a license to display the preprint in perpetuity. It is made available under aCC-BY 4.0 International license.

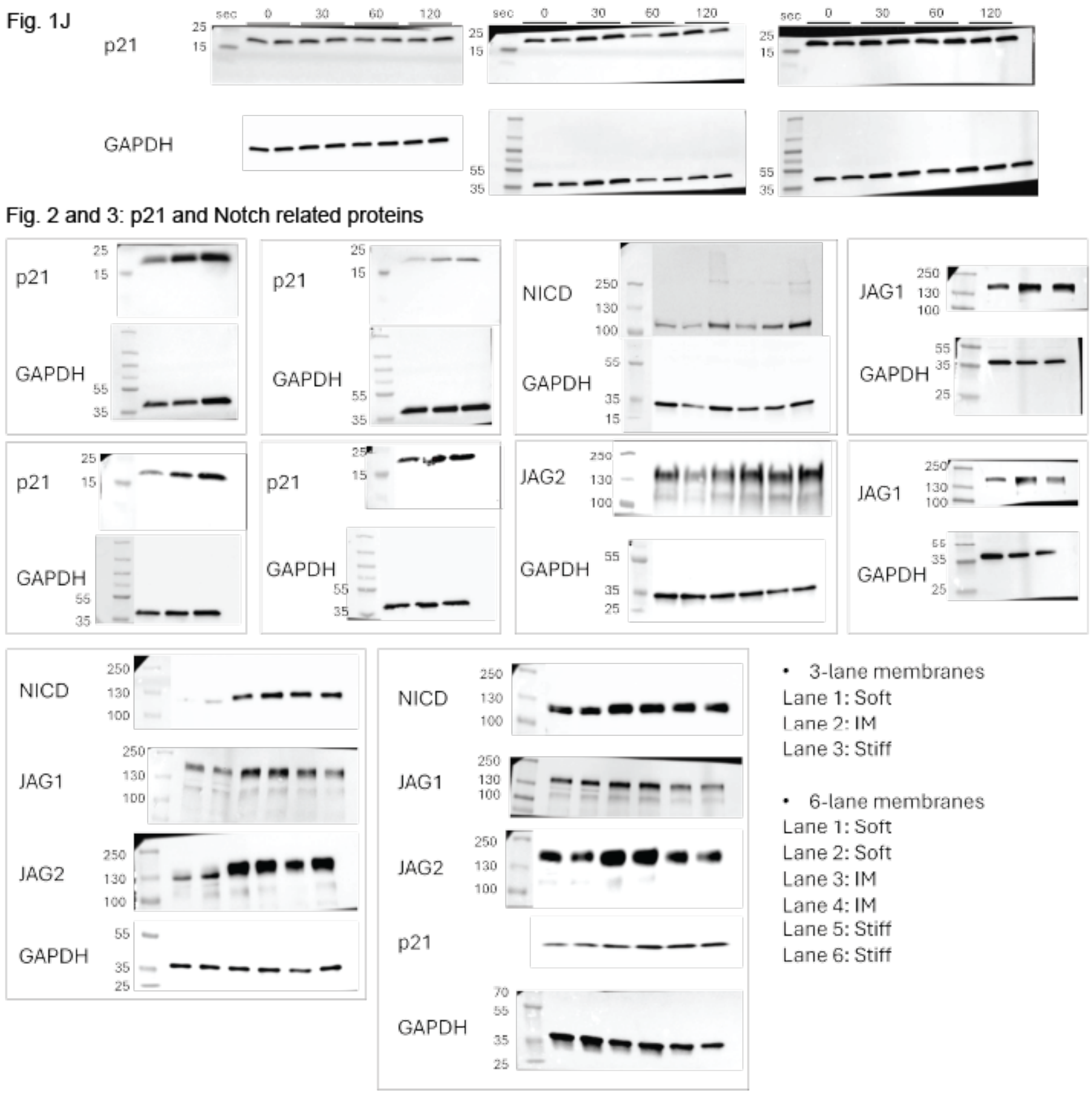

Fig S5. Western blot membranes corresponding to the main figures are shown.

bioRxiv preprint doi: https://doi.org/10.1101/2025.10.05.680536; this version posted October 6, 2025. The copyright holder for this preprint (which was not certified by peer review) is the author/funder, who has granted bioRxiv a license to display the preprint in perpetuity. It is made available under aCC-BY 4.0 International license.

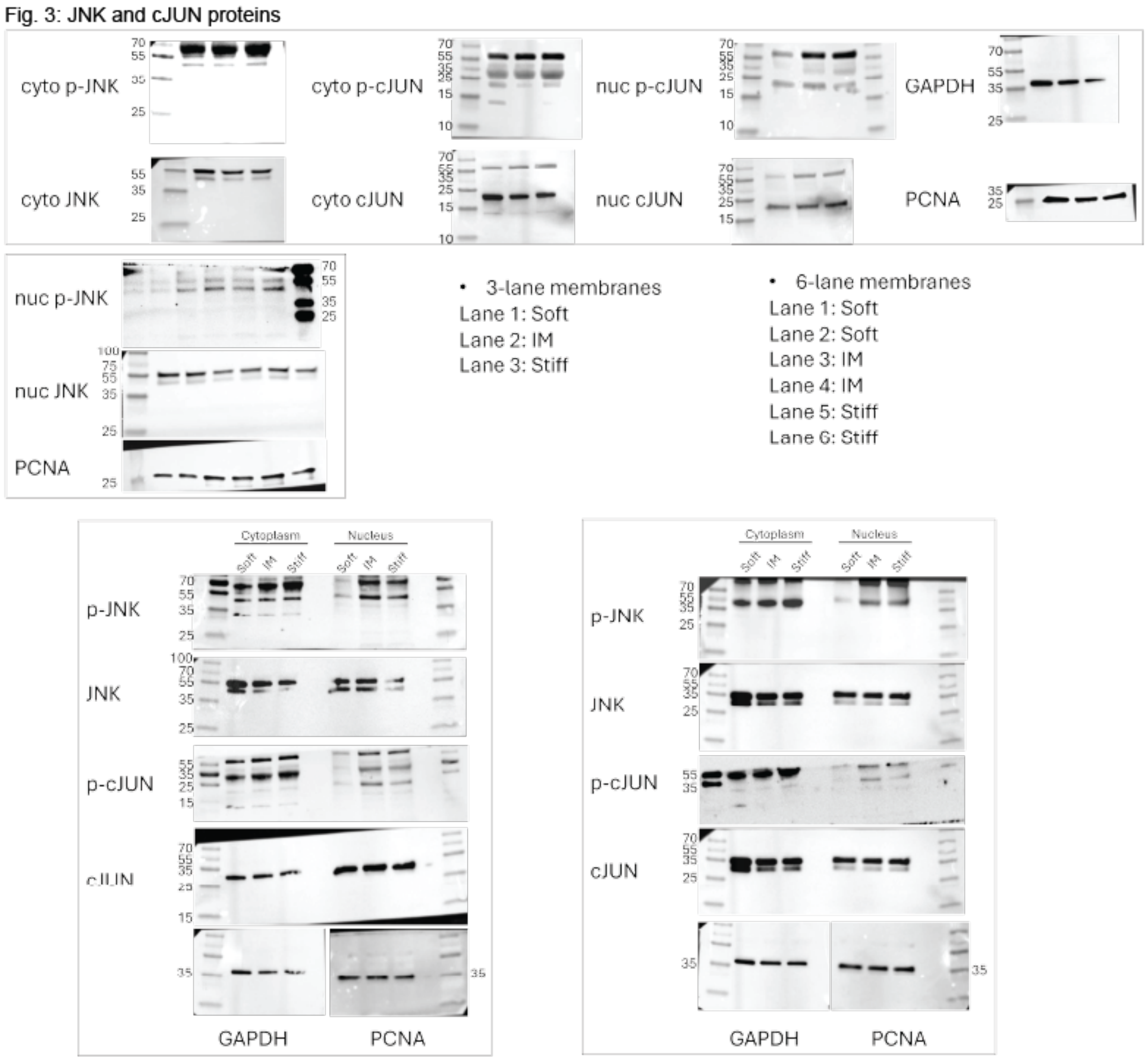

Fig S5 (continued). Western blot membranes corresponding to the main figures are shown.
